# Percent body fat estimations in college men using field and laboratory methods: A three-compartment model approach

**DOI:** 10.1186/1476-5918-7-7

**Published:** 2008-04-21

**Authors:** Jordan R Moon, Sarah E Tobkin, Abbie E Smith, Michael D Roberts, Eric D Ryan, Vincent J Dalbo, Chris M Lockwood, Ashley A Walter, Joel T Cramer, Travis W Beck, Jeffrey R Stout

**Affiliations:** 1 Department of Health and Exercise Science, Metabolic and body composition laboratories, University of Oklahoma, Norman, OK, USA; 2 Department of Health and Exercise Science, Biophysics laboratory, University of Oklahoma, Norman, OK, USA; 3 Department of Health and Exercise Science, Applied biochemistry and molecular physiology laboratory, University of Oklahoma, Norman, OK, USA

## Abstract

**Background:**

Methods used to estimate percent body fat can be classified as a laboratory or field technique. However, the validity of these methods compared to multiple-compartment models has not been fully established. The purpose of this study was to determine the validity of field and laboratory methods for estimating percent fat (%fat) in healthy college-age men compared to the Siri three-compartment model (3C).

**Methods:**

Thirty-one Caucasian men (22.5 ± 2.7 yrs; 175.6 ± 6.3 cm; 76.4 ± 10.3 kg) had their %fat estimated by bioelectrical impedance analysis (BIA) using the BodyGram™ computer program (BIA-AK) and population-specific equation (BIA-Lohman), near-infrared interactance (NIR) (Futrex^® ^6100/XL), four circumference-based military equations [Marine Corps (MC), Navy and Air Force (NAF), Army (A), and Friedl], air-displacement plethysmography (BP), and hydrostatic weighing (HW).

**Results:**

All circumference-based military equations (MC = 4.7% fat, NAF = 5.2% fat, A = 4.7% fat, Friedl = 4.7% fat) along with NIR (NIR = 5.1% fat) produced an unacceptable total error (*TE*). Both laboratory methods produced acceptable *TE *values (HW = 2.5% fat; BP = 2.7% fat). The BIA-AK, and BIA-Lohman field methods produced acceptable *TE *values (2.1% fat). A significant difference was observed for the MC and NAF equations compared to both the 3C model and HW (*p *< 0.006).

**Conclusion:**

Results indicate that the BP and HW are valid laboratory methods when compared to the 3C model to estimate %fat in college-age Caucasian men. When the use of a laboratory method is not feasible, BIA-AK, and BIA-Lohman are acceptable field methods to estimate %fat in this population.

## Background

Accurate assessment of body composition is necessary in order to monitor obesity class, nutritional status, training outcomes, and general health [1]. Validated laboratory methods, such as hydrostatic weighing (HW), and multiple compartment models, like the three-compartment (3C) model, are impractical to use in large population studies. Specifically, Wang et al. [2] concluded that the 3C model of Siri [3] was superior to HW using the Brozek et al. [4] equation to estimate percent body fat (%fat) when compared to the six-compartment model, while Fuller et al. [5] determined that the precision between the four-compartment (4C) and 3C model did not differ. However, some discrepancy exists when comparing HW to the multiple-compartment model in adult men [2,6]. Nonetheless, both multiple-compartment models and HW entail greater facility requirements and are more costly compared to more convenient field methods, such as anthropometric equations, near-infrared interactance (NIR), and bioelectrical impedance analysis (BIA).

When assessing body composition, NIR and BIA are appealing field methods due to the safety, noninvasiveness, and speed of administration when compared to laboratory techniques that require some risk, expensive equipment, and trained personnel [7]. An alternative field method to NIR and BIA is the use of anthropometric measurements to estimate body density (BD) and %fat. Currently, the United States Military uses circumference measurements to estimate the %fat of soldiers and recruits. Specifically, three equations using neck and waist circumferences were developed based on HW for the Marine Corps (MC), Navy and Air Force (NAF), and the Army (A). In 1997, Friedl and Vogel [8] cross-validated these three equations with dual-energy X-ray absorptiometry (DXA) and developed a new equation (Friedl) based on their findings. However, no one has validated these equations for estimating %fat against a multi-compartment criterion method. Nonetheless, if the use of military circumference-based equations is valid for estimating %fat in college-age men, then this could be another rapid and non-invasive method of determining %fat. Therefore, if acceptable agreement is found to exist between established laboratory methods, like the 3C model and field methods, such as NIR, BIA, and circumference-based equations, these field methods could provide potential alternatives to cumbersome laboratory methods.

The purpose of this study was to compare %fat estimations between laboratory methods [Air Displacement Plethysmography via the BOD POD^® ^(BP) and HW], newly unvalidated methods and devices (NIR (Futrex^® ^6100/XL), BIA-AK (RJL Quantum II)), military circumference-based equations and the population-specific BIA-Lohman equation to the 3C model %fat values in college-age Caucasian men.

## Methods

### Participants

Thirty-one Caucasian men volunteered to participate in the study (Table 1). All body composition measurements were performed on the same day following a 12-hour fast (*ad libitum *water intake was allowed). HW was performed last while all prior body composition measurements were performed in no particular order. The subjects were also instructed to refrain from exercising for at least 12 hours prior to testing. The purpose of the study and a description of the testing protocol were explained to each subject. Additionally, the study was approved by The Institutional Review Board for Human Subjects. Written informed consent was obtained from each subject prior to testing.

**Table 1:** Description characteristics of the subjects (*n *= 31)

Variable	$\bar{x}$	SD	Range
Age (y)	22.5	2.7	18 – 33
Body mass (kg)	76.4	10.3	56.88 – 101.7
Height (cm)	175.6	6.3	165.8 – 191.3
Abdominal (cm)	81.3	7.7	69.4 – 105.5
Neck (cm)	38.0	2.2	34.7 – 42.7
BMI	24.8	3.1	20.0 – 31.9

### Hydrostatic Weighing

Body density (BD) was assessed from HW as previously described by our laboratory and others [2,6,9,10]. Residual volume was determined with the subject in a seated position using the oxygen dilution method of Wilmore et al. [11] via a metabolic cart with residual volume software (True One 2400^®^, Parvo-Medics, Inc. Provo, Utah). Subjects completed a minimum of two trials and the average of the closest two trials within 5% was used to represent residual volume.

Underwater weight was measured to the nearest 0.025 kg in a submersion tank in which a PVC swing seat was suspended from a calibrated Chatillon^® ^15-kg scale (Model # 1315DD-H, Largo, Florida). The average of the three highest values (6 to 10 trials) was used as the representative underwater weight. Percent body fat was calculated using the revised formula of Brozek et al[4]. Previous test-retest reliability data for HW from our laboratory indicated that, for 11 young adults (24 ± 2.4 yr) measured on separate days, the ICC was 0.99 with a *SEM *of 0.8% fat and 0.002729 g·cc^-1 ^for BD.

### Air Displacement Plethysmography (BOD POD^®^)

Before each test, the BP was calibrated according to the manufacturer's instructions with the chamber empty using a cylinder of known volume (49.558 L). The subject, wearing tight fitting compression shorts and a swimming cap, entered and sat in the fiberglass chamber. The BP was sealed, and the subject breathed normally for 20 seconds while body volume was measured. Next, the subject was connected to a breathing tube internal to the system to measure thoracic gas volume. The subject resumed tidal breathing cycles; a valve in the circuit caused a momentary occlusion of the airway, during which the subject gently "puffed". This effort produced small pressure fluctuations in the airway and chamber that were used to determine thoracic gas volume. This value was used to correct body volume for thoracic gas volume and percent body fat was calculated from BD using the revised formula of Brozek et al[4]. All BP measurements were performed by a BOD POD^®^-certified investigator who had previously demonstrated a *SEM *of 0.48% fat.

### Near-Infrared Interactance

The Futrex^® ^6100/XL was used to measure the %fat of each subject according to the procedures recommended by the manufacturer (Futrex^®^, Hagerstown, MD). This device emits infrared light of six specific wavelengths (810, 910, 932, 944, 976, and 1,023 nm) into the anterior midline of the *biceps brachii *midway between the *antecubital fossa *and *acromion *process of the right arm. A silicon-based detector then measures the intensity of the re-emitted light, which is expressed as optical density. Percent body fat was estimated using a pre-programmed generalized multiple regression equation that included height, weight, and optical density values. The specific equation used to calculate %fat was not available from the manufacturer. The instrument was calibrated prior to each measurement with the manufacturer-supplied optical standard. Previous test-retest measurements for the Futrex^® ^6100/XL produced a *SEM *of 0.74% fat.

### Bioelectrical Impedance Analysis

BIA analysis was performed using the Quantum II Bioelectrical Body Composition Analyzer following the procedures recommended by the manufacturer (RJL Systems, Clinton Twp, MI). Percent body fat was estimated from the resistance (Ω) and reactance (Ω) values produced by the Quantum II. Briefly, after resting in a supine position for 5 to 10 minutes, resistance and reactance measurements were taken while the subjects lay supine on a table with their arms ≥ 30 degrees away from their torso with their legs separated. Electrodes were placed at the distal ends of the subject's right hand and foot following the manufacturer guidelines. Excess body hair was removed prior to electrode placement. The site (skin) was cleaned with alcohol. The average of two trials within ± 5 Ω was used to represent the subject's resistance (Rz) and reactance (Xc) values. The average of resistance values and the subject's height, weight, sex, and age were entered into a computer program (BodyGram™ Version 1.31, Akern Bioresearch, Pontassieve (Florence), Italy) to estimate %fat (BIA-AK). Additionally, fat free mass was estimated from Lohman's [12] prediction equation (BIA-Lohman) specific to this population and converted to %fat (Table 2). Previous test-retest measurements for BIA-AK produced a *SEM *of 0.68% fat.

**Table 2:** Validation of methods of predicting % body fat compared to the 3C model

	Method	% fat ($\bar{x}$ ± SD)	Slope	Intercept	*CE*	*r*	*SEE*	*TE*	Limits
	3C	16.3 ± 4.7							

Field	MC	13.6 ± 4.1	0.71	6.71	2.69*	0.62	3.78	4.7^†^	5.00, -10.38
	NAF	13.4 ± 4.9	0.55	8.86	2.86*	0.57	3.96	5.2^†^	5.87, -11.60
	A	14.3 ± 4.4	0.60	7.68	1.96	0.57	3.97	4.7^†^	6.44, -10.36
	Friedl	14.4 ± 4.7	0.57	8.01	1.86	0.57	3.94	4.7^†^	6.69, -10.42
	NIR	14.3 ± 4.6	0.50	9.16	1.98	0.49	4.20	5.1^†^	7.30, -11.27
	BIA-AK	17.1 ± 4.5	0.95	0.05	-0.78	0.91	2.00	2.1	4.65, -3.09
	BIA-Lohman	16.0 ± 4.0	1.07	-0.74	0.33	0.90	2.14	2.1	3.83, -4.48

Lab	BP	16.3 ± 5.5	0.75	4.12	0.04	0.86	2.42	2.7	5.35, -5.43
	HW	15.8 ± 4.7	0.87	2.51	0.53	0.86	2.44	2.5	4.31, -5.36

* Represents significance (*p *< 0.0055), ^† ^Represents an unacceptable *TE *(*TE *> 4.0% BF)

*CE *= Constant error, *TE *= Total error, *SEE = *Standard error of estimate, *r *= Pearson product-moment correlation coefficient, Limits = 95% limits of agreement (CE ± 1.96 SD of residual scores (predicted-actual))

% Fat = (body mass - fat free mass)/body mass

3C %fat [3] = [(2.118/body density) - [0.78 × (total body water in L/body mass in kg)] - 1.354] × 100

MC – Marine Corps [18], %fat = (0.740 × abdominal circumference in cm) - (1.249 × neck circumference in cm) + 0.985

NAF – Navy and Air Force [21], % fat = [(4.95/body density) - 4.5] × 100

NAF [20], body density = [0.155 × log (height in cm)] - [0.191 × log (abdominal - neck circumference in cm)] + 1.032

Army [19], %fat = [76.5 × log (abdominal - neck circumference in cm)] - [68.7 × log (height in cm)] + 43.7

Friedl [8], %fat = [0.771 × (abdominal - neck circumference in cm)] - (0.132 × height in cm) + 4.29

NIR = Near-infrared interactance (Futrex 6100/XL)

BIA = Bioelectrical impedance analysis (RJL Systems Quantum II)

AK = Computer-generated from BodyGram™ Version 1.31 (Akern Bioresearch)

Lohman [12], fat free mass (kg) = [0.485 × (height in cm^2^/resistance in Ω)] + [0.338 × (body mass in kg)] + 5.32

BP = BOD POD^® ^[4], %fat = [(4.57/body density) - 4.142] × 100

HW = Hydrostatic weighing [4], %fat = [(4.57/body density) - 4.142] × 100

### Bioimpedance Spectroscopy

Bioimpedance spectroscopy (BIS) was used to estimate total body water (TBW) following the procedures recommended by the manufacturer (Imp™ SFB7, ImpediMed Limited, Queensland, Australia). This technique, explained elsewhere [13], uses a range of frequencies, encompassing both low and high ranges that allow electrical current to pass around and through each cell, and has produced valid estimates of TBW when compared to a criterion method, such as deuterium oxide [9,13,14]. Results from our laboratory have also shown that the BIS device used in the current investigation is a valid measurement tool for determining TBW when compared to deuterium oxide [15]. Furthermore, BIS has been used to assess TBW for multi-compartment equations in previous validation studies [9,16,17]. TBW measurements were taken while the subject lay supine on a table with their arms ≤ 30° away from the torso and legs separated. Electrodes were placed at the distal ends of each subject's right hand and foot following the manufacturer's guidelines. Prior to electrode placement, excess body hair was removed, and the skin was cleaned with alcohol at each site. The average of two trials within ± 0.05 L was used as the representative TBW. Prior to analysis, each subject's height (HT), body mass (BM), age, and sex were entered into the BIS device. The BIS utilized 256 frequencies internal to the device to estimate TBW. Previous test-retest measurements of 11 men and women measured 24 to 48 hr apart for TBW using the Imp™ SFB7 BIS produced a *SEM *of 0.48 L.

### Circumference measurements

Circumference measurements were taken with a Gulick II measuring tape by an investigator who had previously demonstrated a test-retest reliability of *r *> 0.90. Measurements were taken according to the recommendations of Friedl and Vogel [8] at the abdomen (AB) and neck (N). Two measurements within 1% were used to represent AB and N circumferences. The following equations were used to estimate %fat:

MC – Marine Corps [18] = (0.740 × AB) - (1.249 × N) + 0.985

A – Army [19] = [76.5 × log (AB - N)] - (68.7 × log HT) + 43.7

Friedl [8] = [0.771 × (AB - N)] - (0.132 × HT) + 4.29

Body Density and %fat values for the NAF equation were calculated with the following equations:

NAF – Navy and Air Force [20]

BD = (0.155 × log HT) - [0.191 × log (AB - N)] + 1.032

% fat [21] = [(4.95/BD) - 4.5] × 100

### 3C Calculation

The following equation was used to calculate the criterion %fat via the 3C model [3]:

%fat = [(2.118/BD) - (0.78 × TBW/Body Mass (kg)) - 1.354] × 100

The total error of measurement *TEM *for the 3C model was calculated from the following equation [22]:

3C *TEM *= (TBW *SEM*^2 ^+ HW BD *SEM*^2^)^1/2^

3C *TEM *= (0.48^2 ^+ 0.002729^2^)^1/2^

3C *TEM *= 0.1152 %fat

### Data analysis

Validity of %fat estimates (BP, HW, NIR, BIA-AK, BIA-Lohman, MC, NAF, A, and Friedl) was based on an evaluation of predicted values versus the criterion (actual) value from 3C by calculating the constant error (*CE *= actual (3C) – predicted %fat), *r *value, standard error of estimate ($SEE=\text{SD}\sqrt{1-{\text{r}}^{2}}$), and total error ($TE=\sqrt{\sum {\left[\text{predicted}-\text{actual}\right]}^{2}/\text{n}}$) [7]. The mean difference (*CE*) between the predicted (BP, HW, NIR, BIA-AK, BIA-Lohman, MC, NAF, A, and Friedl) and actual (3C) %fat values was analyzed using dependent t-tests with the Bonferroni alpha adjustment (*p *≤ 0.0055) [23]. Additionally, the method of Bland and Altman was used to identify the 95% limits of agreement between the criterion and predicted %fat values [24].

## Results

The 3C model was considered the criterion measure. The average %fat determined by the 3C was 16.3 ± 4.7 %fat. Presented in Table 2 are the results of the validation analyses. Constant error values ranged from 2.86 (NAF) to 0.04 (BP) with significant *CE *differences (*p *≤ 0.005) detected for MC and NAF. The lowest validity coefficient was 0.49 (NIR) and the highest was 0.91 (BIA-AK), while the *SEE *values ranged from 2.00% fat (BIA-AK) to 4.20% fat (NIR). All laboratory methods resulted in acceptable *TE *values (≤ 2.7% fat). Both BIA (BIA-AK, BIA-Lohman) field methods resulted in acceptable *TE *values (≤ 2.1% fat), while all military circumference-based equations (MC, NAF, A, Friedl) and NIR resulted in unacceptable *TE *values (≥ 4.7% fat). Additionally, compared to HW (Table 3), the MC, A, and Friedl, military circumference-based equations produced large but acceptable *TE *values (≥ 3.5% fat and < 4.0% fat). Of the field methods, the 95% limits of agreement were the largest for all circumference-based equations and NIR (≥ 5.00 to -10.36% fat), while the BIA-AK and BIA-Lohman produced smaller limits of agreement (≤ 4.65 to -4.48% fat).

**Table 3:** Validation of methods of predicting % body fat compared to HW

Method		% fat ($\bar{x}$ ± SD)	Slope	Intercept	CE	*r*	*SEE*	TE
HW		15.8 ± 4.7						

Circumference equations	MC	13.6 ± 4.1	0.88	3.85	2.16*	0.78	3.00	3.7
	NAF	13.4 ± 4.9	0.71	6.24	2.34*	0.74	3.20	4.1^†^
	A	14.3 ± 4.4	0.77	4.76	1.43	0.73	3.23	3.6
	Friedl	14.4 ± 4.7	0.74	5.12	1.33	0.75	3.15	3.5

* Represents significance (*p *< 0.0125), ^† ^Represents an unacceptable *TE *(*TE *> 4.0% BF)

*CE *= Constant error, *TE *= Total error, *SEE *= Standard error of estimate, *r *= Pearson product-moment correlation coefficient.

HW = Hydrostatic weighing [4], %fat = [(4.57/body density) - 4.142] × 100

MC – Marine Corps [18], %fat = (0.740 × abdominal circumference in cm) - (1.249 × neck circumference in cm) + 0.985

NAF – Navy and Air Force [21], % fat = [(4.95/body density) - 4.5] × 100

NAF [20], body density = [0.155 × log (height in cm)] - [0.191 × log (abdominal - neck circumference in cm)] + 1.032

Army [19], %fat = [76.5 × log (abdominal - neck circumference in cm)] - [68.7 × log (height in cm)] + 43.7

Friedl [8], %fat = [0.771 × (abdominal - neck circumference in cm)] - (0.132 × height in cm) + 4.29

## Discussion

Both laboratory methods (HW, BP) used to estimate body composition in this study produced acceptable *TE *values (≤ 4% fat), while only the BIA (BIA-AK, BIA-Lohman) methods resulted in acceptable *TE*values (≤ 4% fat) and are acceptable for estimating %fat in college-age Caucasian men. The four military circumferences-based equations (MC, NAF, A, Friedl) and NIR produced unacceptable *TE *values (≥ 4.7% fat) compared to the 3C model. Additionally, the NIR was the only method to produce both unacceptable *TE, *and *SEE *values (≥ 4.2% fat).

### Laboratory Methods

#### Hydrostatic Weighing

Contrary to the findings of Clasey et al. [6], who compared HW to a 4C model (*TE *= 5.8 %fat) in young men, HW produced acceptable *TE *values (*TE *= 2.5%) in the young men in this study. However, in agreement with Clasey et al. [6], HW produced a similar *r *value (*r = *0.85) and a non-significant *CE *(*CE *= 0.90% fat) compared to the current findings (*r = *0.86, *CE *= 0.53% fat). The discrepancies of these findings could be a result of the two-compartment (2C) model used. Clasey et al. [6] used the 2C model of Siri [21], while the current investigation used the Brozek et al. [4] 2C model. Additionally, Visser et al. [25] used the Siri [21] 2C model and found the difference in %fat values from HW and the 4C to be non-significant and < 2% fat. However, Withers et al. [26] used the Brozek et al. [4] equation used in the current study and found a greater *CE *(2.2% fat) than the current investigation (*CE *= 0.53%) compared to the 3C model in both trained and sedentary males. Nonetheless, all three studies concluded that the use of multiple-compartment models that included TBW was more accurate than HW for estimating %fat in men. It appears from the current investigation HW is a valid method for estimating %fat in this population. However, the 95% limits of agreement suggest HW may over-predict %fat by as much as 5.36% fat and under-predict by as much as 4.31% fat. These individual variations are most likely due to the use of a 2C model which does not require an estimate of TBW. Therefore, caution should be used when relying on HW alone as a method to identify %fat in small groups or individuals.

#### Air-displacement plethysmography (BOD POD^®^)

Due to the ease in procedure, speed, and improved subject compliance, BP provides an attractive alternative to HW. Additionally, research has shown that participants prefer the BP over HW [27]. Results of this study demonstrated high validity coefficient (*r *= 0.86) and "excellent" *SEE *(2.42% fat) and "very good" *TE *(2.7% fat) values [7]. Although the BP is a relatively new device, a number of studies have examined the validity when compared to HW in males with contradictory findings [27-30]. However, only limited research is available on the validity of BP in college-age men compared to multiple-compartment models, specifically the 3C model. Nonetheless, the current investigation (*SEE *= 2.42% fat, *r *= 0.86) produced acceptable values similar to those of McCrory et al. [31] who compared BP (*SEE *= 1.81% fat, *r *= 0.96) to HW. To the best of our knowledge, this is the first investigation to compare BP to a multiple-compartment model in college-age males. The current findings suggest, as with HW, that BP is a valid method for estimating %fat in the college-age men. However, the 95% limits of agreement suggest that BP may over-predict %fat by as much as 5.43% and under-predict by as much as 5.35%. These individual variations are most likely due to the use of a 2C model which does not require an estimate of TBW. Therefore, caution should be used when relying on BP alone as a method to identify %fat in small groups or individuals.

### Field Methods

#### Near-Infrared Interactance

The second aim of this study was to examine the validity of the newly-developed NIR device (F6100/XL) employing six wavelengths to estimate %fat. In 1984, Conway et al. [32] measured infrared wavelengths from 700 to 1100 nm and determined that the peak absorption of pure fat occurred at 930 nm and pure water at 970 nm. Based on this research, the Futrex^® ^5000/1000 utilized infrared wavelengths at 940 and 950 nm to measure optical density (OD). However, the updated NIR model used in this investigation (Futrex^® ^6100/XL) employs six different wavelengths at 810, 910, 932, 944, 976, and 1023 nm to estimate %fat [33]. The Futrex^® ^6100/XL has advantages over previous models because the wavelengths encompass the same range as in the original research by Conway et al. [32] (700–1100 nm).

Our results indicated the NIR underestimated an average of 1.98% fat compared to the 3C model. Additionally, the NIR produced unacceptable *SEE *and *TE *values > 4.0% fat. To our knowledge, this is the first complete study to compare the new NIR device to any criterion method. However, other studies have validated earlier models (Futrex^®^5000 and Futrex^®^1000) in males compared to HW [34-38]. These previous studies concluded that neither the F1000 or F5000 were acceptable methods for estimating %fat with reported *TE *or *SEE *values > 4% fat [34-38]. In agreement with all of the past literature on NIR and college-age males, the current findings are unacceptable (*SEE *and *TE *values >4.0% fat). It appears that the six wavelengths utilized in the Futrex^® ^6100/XL compared to the two wavelengths for the Futrex^®^5000 and Futrex^®^1000 did not improve the accuracy for estimating %fat in this population. The large *SEE *(4.20% fat) value suggests that the NIR prediction equation has a large individual deviation from the line of best fit, while the low *r *(0.49% fat) value suggests a poor correlation between actual %fat estimated from the 3C and from NIR. Regardless of *TE*, the NIR produced unacceptable *SEE *(*SEE *> 4.0% fat) and *r *(*r *< 0.80) values. Therefore, the Futrex^® ^6100/XL NIR device is not recommended for use in this population. Future research needs to identify the validity of the Futrex^® ^6100/XL in various populations.

#### Bioelectrical Impedance

Results of previous studies on BIA validity for estimating %fat in college men have been equivocal [37-45]. Single frequency (50-kHz) BIA devices, such as the RJL Quantum II, are based on the work of Thomasett [46], who implemented the use of a low-level electrical current and measured the opposition of flow. This opposition of electrical current through the body is directly related to its composition of water, fat, and lean tissue. Since the electrolytes in water are good conductors of electric current, the opposition of electrical flow can be used to estimate total body water (TBW) and lean body mass, with the assumption that lean body mass has ≅ 73% water [47]. Total body mass and lean body mass are used in the calculation of %fat; however, TBW differences may exist across race, sex, age, and health status. Therefore, body composition experts have suggested using BIA population-specific equations to estimate %fat [7,12]. Lohman [12] developed a population-specific equation for men 18–30 years of age that includes BIA measurement of resistance, height, and weight (BIA-Lohman). A more technical method to assess body composition from BIA uses vectors as described by Piccoli et al. [31] and phase angles as described by Barbosa-Silva and Barros [48], which require BIA-measured resistance and reactance values. The BodyGram™ (Version 1.31, Akern Bioresearch) computer program utilizes resistance and reactance values to estimate %fat (BIA-AK) via vectors and phase angles. However, to the best of our knowledge, no research has validated BIA-AK %fat estimations in healthy college-age Caucasian men.

Previous research has compared BIA devices to HW with contrasting results [37,38,40-45]. The *SEE *(2.00 %fat) values reported in this study for BIA-AK (RJL Quantum II, RJL Systems) compared to the 3C were substantially lower than those reported by other investigators compared to HW [37,38,40-45]. The current *r *value (*r *= 0.91) was greater than most investigations (*r *< 0.84) [37,40-43,45] but similar to the findings of Lukaski et al. [44] (*r *= 0.93). Regarding *TE*, current BIA-AK results (2.1 %fat) are the lowest reported. Though Eckerson et al. [39] reported acceptable fat free-weight values (*TE *= 1.7% fat), the majority of the literature has reported unacceptable *TE *values (*TE *> 4.4) [37,38,40-45]. These discrepancies are most likely due to the variation of devices used and the subsequent equations or methods used to estimate %fat. It appears that the current method used to estimate %fat (BodyGram™ Version 1.31, Akern Bioresearch) is an acceptable procedure to estimate the %fat of college-age men.

The BIA-Lohman population-specific equation produced similar results to the BIA-AK. Past research has produced similar results to those of the other BIA equations when the BIA-Lohman equation was compared to HW. Eckerson [41] (men 22 ± 4 years) found the BIA-Lohman equation to be unacceptable with a *TE *of 4.4% fat. However, the current investigation produced an acceptable *TE *value (*TE *= 2.1% fat).

To the best of our knowledge, there has not been an evaluation of the validity of any BIA method of predicting %fat in college men compared to a multiple-compartment model. Therefore, this is the first investigation showing the strong agreement between the 3C model or any multiple-component model and both BIA-AK and BIA-Lohman in this population. The lower *TE *values in the current investigation compared to past literature comparing BIA to HW are most likely due to the utilization of TBW in the criterion 3C model. The relationship between fat free mass (FFM) and TBW has been well established [47]. The strong correlation between resistance and reactance measured by BIA and FFM measured by BIA could have improved both BIA values in contrast to past literature in which TBW was not estimated. However, in the current investigation, both BIA-AK and BIA-Lohman methods produced larger, but acceptable, *TE *values compared to HW (BIA-AK, *TE *= 3.4% fat; BIA-Lohman, TE = 3.1% fat), suggesting that TBW may increase the accuracy of BIA procedures. Nonetheless, the removal of TBW in the criterion method did not produce unacceptable BIA *TE *values (*TE *< 4.0% fat). Future research is needed to re-evaluate the validity of previously-used equations and BIA devices compared to multiple-compartment models that utilize TBW. Furthermore, it appears that both BIA-AK and BIA-Lohman are acceptable field methods for estimating %fat in college men. However, the 95% limits of agreement suggest both BIA-AK and BIA-Lohman may over-predict %fat by as much as 3.09 and 4.48% fat and under-predict by as much as 4.65 and 3.83% fat, respectively. These individual variations are most likely attributed to deviations in intra-cellular fluid which a single frequency (50-kHz) BIA device cannot detect. Therefore, caution should be used when relying on BIA alone as a method to identify %fat in small groups or individuals.

#### Circumference measurements

The final aim of this study was to examine the validity of military circumference-based equations to estimate %fat compared to both the 3C model and HW. In agreement with Clasey et al. [6] (*CE *= 4.2% fat), who compared a similar circumference-based equation [49] to the 4C model in young men, our results indicated all equations underestimated (CE ≥ 1.86% fat) %fat compared to the 3C model. Additionally, all equations produced unacceptable *TE *values > 4.0% fat, which is similar to the reported *TE *(6.98% fat) value by Clasey et al. [6]. To our knowledge, this is the first study to compare military circumference-based equations to the 3C model. However, the equations used in the current investigation have been previously validated against HW in a similar population [8,42]. The current *SEE *values (*SEE *= 3.79–3.96 %fat) were greater when compared to the 3C than the findings of Friedl and Vogel [8], who compared the MC (*SEE *= 3.27% fat), NAF (*SEE *= 3.13% fat), A (*SEE *= 3.15% fat), and Friedl (*SEE *= 3.10% fat) equations to DXA in male soldiers (< 40 years, mean BM = 77.4 kg, HT = 176.7 cm). Additionally, all the *r *values in the current investigation (*r *≤ 0.62) were less than those reported by Friedl and Vogel [8](*r *≥ 0.80). Furthermore, Kremer et al. [42] compared the NAF equation to HW in Air Force members 18–47 years of age (mean body mass = 87.2 kg, height = 178.5 cm) with contrary findings to the current investigation. Kremer et al. [42] found smaller a *SEE *(3.28% fat) and *TE *(3.54% fat) value and a larger *r *(0.91) value than the present results (*SEE *= 3.96% fat, *TE *= 5.2% fat, *r *= 0.57). Therefore, all four circumference-based equations are not recommended for estimating %fat in college-age men.

It was hypothesized that all four circumference-based equations would produce valid results when compared to HW. Contrary to our hypothesis, only the MC, A and Friedl equations produced acceptable *TE *values (*TE *≤ 3.7). Conversely, all *TE *values were ≥ 3.5% fat, which is sometimes classified as the minimal standard [7]. While the NAF equation was in closer agreement with the findings of Kremer et al. [42] (*TE *= 3.54% fat), the *TE *value was still unacceptable (*TE *= 4.1% fat). Nonetheless, the study by Friedl and Vogel [8] produced comparable *SEE *values for the MC (*SEE *= 3.27% fat), A (*SEE *= 3.15% fat) and Friedl (*SEE *= 3.10% fat) equation to the current investigation compared to HW (MC, *SEE *= 3.00% fat; A, *SEE *= 3.23% fat; Friedl, *SEE *= 3.15% fat). In conclusion, the results of the current investigation suggest that military circumference-based equations are not valid methods to estimate the %fat of college-age Caucasian men and are not recommended for use in this population.

## Conclusion

Both 2C laboratory methods (BP, HW) produced acceptable *TE *values when compared to the 3C model of Siri [3]. However, due to the large limits of agreement, the use of 2C models may not be appropriate when attempting to identify and track %fat in small groups or individuals due to the individual variations of a third compartment [2,6,26]. Wang et al. [2] determined that the 3C model used in the current investigation along with 4C models were some of the best methods to use as criteria %fat estimates compared to the 6C model and concluded these 2C models (BP, HW) may not be appropriate across all age, sex, and disease groups for estimating %fat. However, the current investigation provides data that validate the use of these 2C models (BP, HW) for use in college age males. Thus, caution should be used when utilizing one of these 2C models in a research setting when %fat or other body composition compartments (fat-free mass, fat mass) are being estimated or tracked for small groups or individual college-age Caucasian men.

Limitations of the current study include the estimates of %fat from the 3C model. Since %fat values from the 3C model included measurements from HW, there could potentially be greater agreement between HW and the 3C model. Additionally, multiple-compartment models with greater complexity than the 3C, such as the 4, 5 and 6C models, are better criterion methods, and, thus, should be used to validate the methods included in the current study in this population. Furthermore, TBW measurements were obtained via BIS, which is a valid measure but not a criterion method. The possibility exists that the use of a criterion method, such as deuterium oxide, to measure TBW could influence the values found in the current investigation.

In summary, this study provides original data regarding the validity of laboratory and field methods. Our data suggest BP is an acceptable laboratory method to use when HW or multiple-compartment models are not available or subject compliance is a potential problem. Furthermore, our results indicate that BIA-AK, BIA-Lohman are valid alternatives to estimate %fat when laboratory methods are unavailable for college-age Caucasian men. However, caution should be used when using NIR and military circumference-based equations to estimate %fat in this population and are not recommended.

## Competing interests

The authors declare that they have no competing interests.

## Authors' contributions

JM, ST, JC, TB, and JS participated in the study design and helped draft the manuscript while aiding in data collection. AS, AW, CL, MR, ER, and VD participated in data collection and analysis. Additionally, all authors read and approved the final manuscript.
